# Utilization of cervical cancer screening services and its associated factors in Iran: a case–control study

**DOI:** 10.1186/s13027-023-00496-w

**Published:** 2023-03-11

**Authors:** Sara Dadipoor, Azin Alavi, Zainab Kader, Hadi Eshaghi Sani Kakhaki, Shokrollah Mohseni, Nahid Shahabi

**Affiliations:** 1 Mother and Child Welfare Research Center, Hormozgan University of Medical Sciences, Bandar Abbas, Iran; 2 Social Determinants in Health Promotion Research Center, Hormozgan Health Institute, Hormozgan University of Medical Sciences, Bandar Abbas, Iran; 3 The Centre for Interdisciplinary Studies of Children, Families and Society, Faculty of Community and Health Sciences, University of the Western Cape, Bellville, South Africa

**Keywords:** Cervical cancer screening, Knowledge, Access, Suburbs, Iran

## Abstract

**Background:**

Considering the high incidence rate of cervical cancer (CC) in Iran, screening is an effective way of reducing the impact of the disease due to early detection. Thus, the recognition of the factors affecting the use of cervical cancer screening (CCS) services is essential.The present study aimed to determine the associated factors of CCS in women living in the suburbs of Bandar Abbas in the south of Iran.

**Methods:**

The present case–control study was conducted between January and March 2022 in the suburban areas of Bandar abbas. Two hundred participants were assigned to the case group and 400 participants were assigned to the control group. A self-constructed questionnaire was used to collect the data. This questionnaire contained demographic information, reproductive information, knowledge of CC, knowledge of CCS and access to the screening. Univariate and multivariate regression analyses were run to analyze the data. The data were analyzed in STATA 14.2 at a significance level of *p* < 0.05.

**Results:**

The mean and standard deviation of participants’ age in the case group was 30.33 ± 4.892, and in the control group was 31.35 ± 6.149. The mean and standard deviation of knowledge in the case group was 10.21 ± 1.815 and in the control group was 7.24 ± 2.447. The mean and standard deviation of access was 43.72 ± 6.339 in the case and 37.17 ± 4.828 in the control group. The results of multivariate regression analysis showed the following factors increased the odds of CCS: knowledge (OR medium = 18.697, OR high = 13.413), access (OR medium = 4.028, OR high = 8.126), being married (OR = 3.193), being educated (OR diploma = 2.587, OR university degree = 1.432), middle and high SES (OR Middle = 6.078, OR Upper = 6.608), and not smoking (OR = 1.144). Also, women's reproductive status, including history of sexually transmitted diseases (OR = 2.612), use of oral contraceptives (OR = 1.579), sexual hygiene (OR = 8.718).

**Conclusions:**

In the light of the present findings, it can be concluded that besides increasing suburban women’s knowledge, their access to screening facilities should be improved. The present findings showed the need to remove the barriers to CCS in women of low SES to increase the rate of CCS. The present findings contribute to a better understanding of factors involved in CCS.

## Background

cervical cancer (CC) is the fourth most prevalent cancer and the fourth leading cause of cancer-related mortalities in women, accounting for 604,000 new cancer cases and 342,000 mortalities worldwide in 2020 [1]. The incidence of CC in Iran is on the rise [2]. WHO cervical cancer country profiles reported 2.5 for Crude CC incidence per 100 000 women (2020) and 0.61 for Cervical cancer mortality-to-incidence ratio (2020) [3]. Persistent human papillomavirus infection is a necessary cause of cervical cancer and geography, traditional practices and beliefs, screening levels, socioeconomic status (SES), access to health care services, public awareness, use of oral contraceptives, smoking, and HIV infection are risk factors of CC [4].

Due to the increasing rate of this disease, the World Health Organization (WHO) has announced a call to eliminate CC through vaccination and screening worldwide, which is defined as an incidence rate of less than 4 per 100,000 women annually [5]. CC elimination in low- and middle-income countries takes significantly longer than high-income countries. It is predicted that high HPV vaccination coverage of girls can lead to cervical cancer elimination in most low- and middle-income countries by the end of the century [6]. Also, screening women deprived of HPV vaccination accelerates the elimination of cancer [6, 7].

Despite the availability of the Gardasil vaccine in Iran [8], the high cost of HPV vaccination has restricted the use of this vaccine [9]. Nevertheless, given the long pre-onset period of CC, screening will reduce mortality in the intervening years and offer prevention to women who have missed the opportunity for prophylactic vaccination as they into screening age [10]. The results of a national CCS program in Sweden showed that the risk of CC was higher in women who were not screened than in those who were screened earlier [11]. Landy reported that CCS prevents 83% of non-localized cancers and increased screening coverage is necessary to reduce the incidence of CC as far as possible [12]. However, most of the high budgets for this disease in Iran include treatment [13].

In Iran, the Pap test is performed for CCS programs based on the guidelines of the Ministry of Health, Treatment and Medical Education at 3–5-year intervals [14]. 4 in 10 Iranian women have been screened for cervical cancer in the last 5 years according to Cervical cancer Iran (Islamic Republic of) 2021 country profile [3]. A large number of women do not participate in the screening program, and this low participation rate is more significant in slums and suburban areas [15]. Research suggests that women’s knowledge of CC and their reception of the screening programs are low in suburban areas [16, 17]. The major barriers to participation in the screening programs are the deficiencies of the healthcare system, the difficulty of access, low health literacy and socio-cultural factors [15]. A systematic review reported the most common barriers to performing the Pap test were embarrassment, fear of the screening practical method or the test result, living in remote or rural areas, and limited health resources/infrastructure [18].

Considering the problems caused by CC, it is essential to know the factors affecting the use of CCS services. A review of the related literature showed that most of the previous studies have been conducted to evaluate the success of the screening program [11, 19, 20], or the factors affecting screening in areas other than the suburban [21–23]. Few studies have been conducted on slum areas in other countries [16, 17]. So far, no study in the suburban areas of southern Iran has addressed the factors affecting CCS. Therefore, considering the social and geographical inequalities of CC [24], and the low level of knowledge and differences in screening in different geographical areas of Iran [25], it is necessary to conduct a study emphasizing knowledge of and access to screening facilities in suburban areas. The recognition of the role of these factors can enable health policymakers to consider effective strategies to lower inequalities and increase CCS coverage.

Hypothesis: knowledge and access to screening services are related to CCS.

Aim: Determining the associated factors of CCS in women living in the suburbs of Bandar Abbas in the south of Iran.

## Methods and materials

### Study design and population

The present case–control study was conducted between January 2022 and March 2022 among 18–49-year-old women living in the suburban areas on Bandar Abbas in Hormozgan Province. This city is located in the 27.19 latitude and 56.28 longitude and the height of 9 m above the sea level. Bandar Abbas has a population of 352,173 making it the largest city in Hormozgan.

The coastal city of Bandar Abbas has commercial affairs with the countries of the Persian Gulf and is considered a destination for job seekers in Iran. Their arrival has significantly increased the population and led to the emergence of slums, which has also caused social and health problems [26].

In Iran, the breast and CCS is currently done as a health program in the middle-aged population of 30 to 59 years of age in national healthcare centres [27]. Women’s participation in screening programs is voluntary. Of note is that the screening test is free of charge in comprehensive healthcare centres. However, screening services are not free in midwifery centres and gynecology clinics and women need to pay for that.

In health care centers, the health information about households, including the status of women's CCS, is recorded in the “Sib” integrated national health system, also, Pap test sampling is performed by the midwives.

In this study, women in the case group had performed the CCS regularly in the past 3–5 years, while women in the control group had not performed the CCS in the past 3–5 years. To ensure the absence of CCS in the control group, the women were asked about when last they had screening done and their status was checked on “Sib” to verify.

### Inclusion criteria

1. Women aged between 18 and 49 years
2. Residing in areas covered by health service centers in the suburbs: Payambar Azam, Chahestaniha, Islamabad, Soro, Katbi and Tawheed streets
3. Conducting CCS regularly within the past 3–5 years in the case group and not performing CCS during the past 3–5 years in the control group
4. Providing an informed consent to participate in the study
5. Being able to read and write

### Exclusion criteria

1. Incomplete questionnaires
2. Having family history of CC (family history of CC increases the knowledge of CC and encourages the performance of Pap test [28])

### Sampling and sample size

A multivariable logistic regression analysis was run with 20 variables. As a general rule in logistic regression models, 10 subjects are needed for each independent variable within the model. Therefore, for each research group, the minimum sample size needed was estimated at 200. To increase the statistical efficiency, the sample size for the control group was twofold (i.e., 400).

**Fig. 1:**
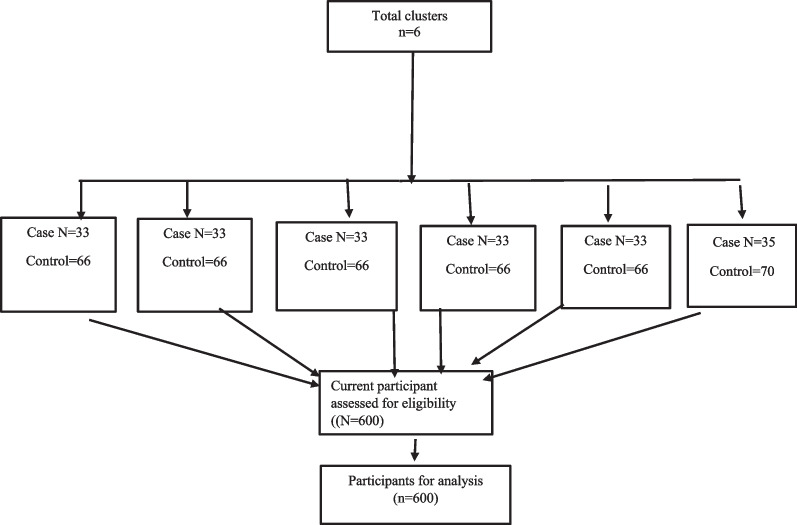
Sampling flowchart

The sampling method comprised of four steps. Firstly, six comprehensive health service centers in Payambar Azam, Chahestaniha, Islamabad, Soro, Katbi and Tawheed were selected. This health service centers covered the suburban areas. Secondly, the researchers went to all six suburban centers to prepare a list of the women receiving services. Thirdly, based on the information recorded in Sib in each center, the list of women who had been screened for CC within the past three years were extracted and then they were separated from other women who had not performed the screening regularly in the past three years. Lastly, through simple randomization, 33 women were selected as the case group from the list of screened women in each center and 66 women were selected as the control group from the second list. In Tawheed health center, which covered more women, 35 participants were selected as the case and 70 as the control (Fig. 1).

### Instrument

The data collection instrument was a tripartite researcher-constructed questionnaire based on a comprehensive review of the related literature [22, 29]. The data were collected as self-reports.

Part 1: Demographic information and reproductive information: Demographic information included age, marital status, educational level, occupation, socio-economic status, and smoking status. Reproductive information included the age of marriage, parity, history of sexually transmitted diseases, use of oral contraceptives and personal sexual health.

Part 2: The knowledge questions were tested with three choices, “yes”, “no”, and “I don't know”. They were scored 1, 0 and 0, respectively. There were 15 questions including those related to CC with 10 questions (#1–10), such as “A family history of CC can increase the probability of this disease”, and questions related to the Pap test, with 5 questions (#11–15), such as “Performing a pap test is necessary only for women who experience abnormal bleeding”. Items 2, 8, 10, 12 and 14 were reversely scored. In these items, a NO answer was considered correct and a YES answer as incorrect. Thus, one who answered NO received the score 1 and he who answered YES or DON’T KNOW received 0. The classification of knowledge questions was done based on the cut-off point by dividing 15 total scores into three categories, as follows: 0–5 low, 6–10 medium, and 11–15 high.

Part 3: Questions related to access to screening services: These include ten questions to be rated on a 5-point Likert scale (very often, often, less frequently, rarely, never) to be scored between 1 and 5. An example item is "To what extent does the distance from the laboratory to deliver the sample (Pap test sample) affect your decision to screen for CC?" The classification of access questions based on the cut-off point based on scores was as follows: 10–23 referred to low, 24–37 referred to medium, and 38–50 referred to high.

### Data quality management

The validity of the present researcher-constructed questionnaire was tested using content validity. The first draft was provided to a panel of experts (5 health education and promotion specialists and 5 obstetrician-gynaecologists) to check the readability, simplicity, relevance and significance of the content. Their comments were used to revise the questionnaire.

Before the main data collection phase, the questionnaire was piloted with 30 participants older than 18 years of age, and their feedback was used to improve the readability and organization of the content. These respondents were selected from six different areas. Their comments were used to revise the initial draft and then these respondents were excluded from the main data collection phase. Its reliability was approved, based on the Cronbach’s alpha test result. The 2-week test–retest with 30 participants was used to check the reliability of the instrument.

Next, the scores of the first and second tests were compared. If the correlation coefficient of the first and second tests in each part was higher than 0.7, the construct was reliable. To calculate the agreement between test and retest, the ICC index was estimated. Thus, to estimate the agreement between the mean test scores and the mean retest scores of knowledge, a CC of 0.94 was estimated. For the access questionnaire, Cronbach's alpha was estimated at 0.876 with an ICC of 0.843. Therefore, the reliability was approved. As the researcher was physically present when the data were collected, there were no defective questionnaires.

### Data collection

After obtaining ethical approval and the required permissions to conduct the study from hormozgan university of medical sciences, the researchers went to the selected health centers and collected the data. A researcher fluent in the local language together with a health care provider of each area visited the participants (case and control groups) that met the inclusion criteria. Having completed the consent form, the participants were asked to complete the questionnaires. The participants in face-to-face meetings with the researcher completed structured questionnaires which took approximately ten minutes to complete. The women could also complete the questionnaire surveys at home at their convenience and return them later to the researcher.

### Ethical considerations

In order to collect the data, the researcher visited the comprehensive health service centers with an official introduction letter from the University describing the study and data collection needs. Moreover, the researcher introduced himself to all participants and explained the purpose of study in a clear and concise manner that was easy to understand. This was followed by obtaining written consent. Participation in this study was completely voluntary and anonymous. The confidentiality of information was preserved. This study was approved by the Ethics Committee of Hormozgan University of Medical Sciences (#IR.HUMS.REC.1401.228).

### Data management and analysis

The case and control interval variables included the knowledge of CC, knowledge of pap test and access. Descriptive statistics (mean and standard deviation) were used for non-interval variables (age, marital status, age of marriage, education, occupation, socio-economic status, parity, history of sexually transmitted diseases, use of oral contraceptives, personal sexual hygiene and smoking status). These were described as frequency and percentage. Independent-samples T-test was run to compare the mean scores of the model constructs between the two groups (one with and the other without the screening program). To relate the screening behavior with demographic variables, fertility variables, knowledge and access, univariable logistic regression was first performed, and then the variables with a *p*-value > 0.025 in the univariable logistic regression analysis were used in multivariable logistic regression analysis. Since age and occupation were not significant in univariate regression, they did not enter the multivariate logistic regression. The data were statistically analyzed in STATA 14.2 and a *p*-value < 0.05 was considered as statistically significant.

## Results

### Sample characteristics

In this study, 200 and 400 participants were included, respectively, in the case and control groups. The mean and standard deviation of age in the case group was 30.33 ± 4.892 and 31.35 ± 6.149 in the control group. Most participants in both groups were in the 30–39-year age group (51.5% as the case and 55.0% as the control). 98.5% of the case and 93.3% of the control groups were married. Most participants held a diploma (49.5% of the case and 64.5% of the control).

Of total 83.5% of the case and 69.8% of the control were of a middle SES. 68% of the control and 48.5% of the case groups had 0–2 childbirths. 86% of women in the case group and 73.3% of those in the control group had a history of sexually transmitted diseases. Also, 96% of women in the control group and 67.5% of the case group did not observe personal sexual hygiene. The knowledge score of 28.2% of women in the control group and 50.5% of the case group was at an average level. In terms of access, 55.5% of the case group and 20.5% of the control had high access. Other demographic information is recorded in detail in Table 1.

**Table 1:** Research participants' sociodemographic and reproductive information

Sociodemographic information				Reproductive information			
Variable	Categories	Case N (%)	Control N (%)	Variable	Categories	Case N (%)	Control N (%)
Age	Mean ± SD	30.33 ± 4.892	31.35 ± 6.149	Age of marriage	15–20	99(49.5)	292(73.0)
	18–29	76(38.0)	159(39.8)		21–25	96(48.0)	83(20.8)
	30–39	110(55.0)	206(51.5)		26–33	5(2.5)	25(6.3)
	40–49	14(7.0)	39(8.8)	Parity	0–2	97(48.5)	216(54)
Marital status	Divorced/widowed	3(1.5)	27(6.8)				
	Married	197(98.5)	373(93.3)		3–5	103(51.5)	184(46
Educational level	Elementary/secondary	12(6.0)	100(25.0)	History of sexually transmitted diseases	No	28(14.0)	107(26.8)
	Diploma	129(64.5)	198(49.5)		Yes	172(86.0)	293(73.3)
	University	59(29.5)	102(25.5)	Oral contraceptives	No	70(35.0)	194(48.5)
occupation	Housewife	188(94.0)	363(90.8)		Yes	130(65.0)	206(51.5)
	Employed	12(6.0)	37(9.3)	Personal sexual hygiene	No	135(67.5)	384(96.0)
SES	Lower	8(4.0)	91(22.8)		Yes	65(32.5)	16(4.0)
	Middle	167(83.5)	279(69.8)	Knowledge	Low	6(3.0)	204(51.0)
	Upper	25(12.5)	30(7.5)		Medium	101(50.5)	113(28.2)
Smoking status	Yes	62(31.0)	219(54.8)		High	93(46.5)	83(20.8)
	No	138(69.0)	181(45.3)	Access	Low	23(11.5)	164(41.0)
					Medium	66(33.0)	154(38.5)
					High	111(55.5)	82(20.5)

The comparison of mean knowledge and access scores between the two groups (the screened and the unscreened groups) is depicted in Table 2. The case group obtained higher scores and the between-group difference was statistically significant (*p*-value < 0.001).

**Table 2:** Mean knowledge and access cores in the two research groups

Variable	Score range	Case Mean ± SD	Control Mean ± SD	*p*-value
Knowledge of CC	10–0	6.17 ± 1.586	4.50 ± 1.754	< 0.001
Knowledge of pap test	5–0	4.03 ± .544	2.74 ± 1.006	< 0.001
Total knowledge score	0–15	10.21 ± 1.815	7.24 ± 2.447	< 0.001
Access	50–10	43.72 ± 6.339	37.17 ± 4.828	< 0.001

**Fig. 2:**
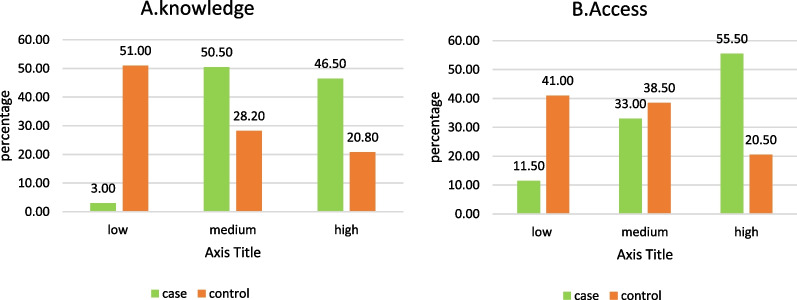
Knowledge and access scores in the case and control groups

Figure 2 shows that approximately half of participants in the case group (46.50%), and 20.80% in the control group had a high knowledge. As for access, 55.50% and 20.50% of the case and control groups, respectively, had high access to health care services.

In univariate regression analysis, no statistically significant difference was found between the case and control groups in the odds of screening in different age groups. The odds of screening in the past 3–5 years were 4 times higher in the married group compared to the divorced/widowed group. Those with a medium and high level of knowledge were 30 and 38 times more likely to show the screening behavior than those with low knowledge. Those with a medium and high levels of access had, respectively, 3 and 9 times as high odds of showing the screening behavior (Table 3).

**Table 3:** Univariate and multivariate logistic regression analyses for CCS

Variable	Categories	Univariate				Adjusted			
		*p*-value	OR	95% C.I. for EXP(B)		*p*-value	OR	95% C.I. for EXP(B)	
				Lower	Upper			Lower	Upper
Age	18–29	.040							
	30–39	0.554	1.117	0.781	1.598				
	40–49	0.606	0.837	0.425	1.648				
Marital status	Divorced/widowed								
	Married	.011	4.753	1.424	15.864	.000	3.193	2.194	5.647
Age of marriage	15–20	.000							
	21–25	.000	3.411	2.352	4.948	.087	4.038	.818	9.931
	26–33	.295	.590	.220	1.583	.465	.899	0.462	3.332
Educational level	Elementary/secondary	.000							
	Diploma	.000	5.429	2.867	10.281	.028	2.587	1.629	7.000
	University	.000	4.820	2.444	9.507	.007	1.432	1.127	4.558
Occupation	Housewife								
	Employed	.174	1.597	.813	3.135				
Economic	Lower	.000							
	Middle	.000	6.809	3.223	14.382	.000	6.078	2.363	15.633
	Upper	.000	9.479	3.867	23.239	.001	6.608	2.114	20.655
Parity	0–2								
	3–5	0.203	1.246	0.874	1.776	.104	1.968	.752	2.943
History of sexually transmitted disease	No								
	Yes	.001	2.243	1.421	3.542	.003	2.612	1.384	4.928
Oral contraceptives	No								
	Yes	.002	1.749	1.232	2.483	.023	1.579	1.161	2.328
Personal sexual hygiene	No								
	Yes	0.000	11.556	6.463	20.661	.000	8.718	6.387	12.498
Smoking status	Yes								
	No	.000	2.693	1.882	3.855	.000	1.144	1.089	2.233
Knowledge	Low	.000							
	Medium	.000	30.389	12.924	71.460	.000	18.697	11.598	27.908
	High	.000	38.096	16.055	90.397	.000	13.413	10.064	19.277
Access	Low	.000							
	Medium	.000	3.056	1.811	5.156	.005	4.028	1.510	10.748
	High	.000	9.652	5.730	16.259	.000	8.126	3.061	21.570

Multivariate regression analysis in Table 3 showed that the odds of performing screening behavior in married participants were 3 times as high as the unmarried participants. The odds of screening behavior among those holding a diploma and a university degree were, respectively, 2 and 1.4 times higher than those with an elementary school education. The odds of performing screening behavior were 6 times higher in both middle and high SES groups. The odds of screening behavior were 1.1 times higher than in non-smoking women as smokers. Compared with women with a low level of knowledge, the odds of performing screening behavior were 18 and 13 times higher in the medium and high levels, respectively. In the medium and high access group, the odds of screening behaviors were 4 and 8 times higher than the low access group.

## Discussion

The present study was conducted to explore the role of demographic variables, knowledge and access to CCS of women living in the suburbs of Bandar Abbas city. Based on the results of multivariate regression analysis, the demographic factors marital status, education, SES, parity, history of sexually transmitted diseases, oral contraceptives, personal sexual hygiene and smoking status showed to be significantly correlated with CCS. Also, knowledge and access were positively and significantly correlated with the CCS behavior.

As the present findings showed, approximately half of the case group and less than a third of the control group had a high level of knowledge. The mean CC and Pap test knowledge scores of the case group were higher than the control. Researchers reported different levels of knowledge in different studies. Consistent with our findings in many studies, the level of knowledge was low in women who had not performed the screening [16, 17]. Contrary to our findings, a study in Saudi Arabia in 2021 reported that the participants’ knowledge of Pap test was between medium and high [30]. The possible cause of this discrepancy could be the difference in the participants’ demographic variables in the above-mentioned studies, for example, 83% of the female participants in Alissa's study had a university degree, but the present participants had a lower level of education.

The current findings showed a positive correlation between knowledge and screening. Thus, those with a higher knowledge had higher odds of performing the screening behavior. This finding was in line with the results of other studies [31, 32]. Evidence suggests that low knowledge is associated with the behavior of not performing the screening [33]. Knowledge is a major predictor of health behaviors and an effective factor in performing screening methods [34]. This finding shows that awareness-raising can be useful for women to perform the CCS. Therefore, there is a need to increase knowledge as well as develop and implement policies in this regard, especially in low resourced areas. There is a need for authorities to prioritize the promotion of women's awareness.

This study revealed that access was positively correlated with CCS. A higher access was associated with higher odds of screening. More than half of the case group and less than a third of the control group had high access to screening facilities. In other words, the mean access scores of women in the case group were higher than the control group. In this study, access included different aspects, such as affordability for the Pap test, the physical distance from the center, the crowd, and the presence of skilled staff in the health center. In line with our findings, several studies reported these mentioned factors to be effective in CCS [31, 35, 36]. Muluneh et al. (2019)reported that a proper timing for women not to delay was significantly correlated with CCS services [37]. Also, Nõmm in Estonia reported that interruption in health insurance was associated with a 23% increase in the risk of disease [38]. The gender of healthcare workers performing the screening has been mentioned as a barrier to performing the screening by women in a number of studies [36, 39]. According to the rules in Iran, the one who performs the Pap test should be a woman. Thus, there was no problem raised by the performer’s gender in the Iranian context. It appears that access is a key factor in increasing the CCS rate. It seems that economic and social inequalities in deprived areas comprise one of the reasons for the problems of accessing screening services. A study in Iran showed that the number of doctors in deprived areas is less than the standards compared to the covered population [40]. Another study emphasized the focus of health policy makers on the access and the distribution of resources based on health needs [41]; therefore, political and organizational efforts to strengthen the budget and human resources in these areas can be useful to improve screening behavior. This issue should be included in health policy makers’ agenda to reduce the burden of this disease as much as possible.

This study showed that demographic variables such as being married, education higher than elementary/secondary school, middle and high SES, and not smoking were significantly correlated with CCS. This correlation was not found in different age groups and occupations.

Arguably, the features of a healthy life style such as no smoking can predict a regular CCS. In a number of studies, an unhealthy life style marked by cigarette smoking, alcohol consumption, obesity and overweight showed to be associated with less participation in CCS programs [42, 43]. It seems that those living an unhealthy life style tend more to show unhealthy behaviors.

In line with the present findings, some studies showed that the screening rate was higher in married, educated, high-income women living in urban areas and those who were employed [21, 22, 33, 44, 45]. In line with the present study, Radha Acharya Pandey did not find any statistically significant correlation between age and CCS [46], but in a number of studies, age was considered as an effective factor in performing CCS [22, 33]. Also, contrary to our findings, Okunowo stated that higher education was not associated with screening, but was significantly associated only with increased knowledge of CC and knowledge of Pap test [28]. One reason for this contradiction could be the type of demographic structure of the present study. For example, considering the conditions governing women in the suburbs of Bandar Abbas, even with high education, most women are not employed. Thus, the factors affecting CCS should be determined according to different demographic variables. CCS is particularly essential in the suburban areas that accommodate the deprived population.

The present study showed a statistically significant relationship between reproductive variables, a history of sexually transmitted diseases, use of oral contraceptives, observing personal sexual hygiene and performing the CCS. Besides being correlated with the CCS behavior, the above-mentioned variables were also significantly correlated with cancer [34]. It can be argued that women during visit the healthcare centers for regular examination, getting free contraceptives, prenatal care and sexual care services, can be informed about screening. In line with our results, the factors influencing CCS in several studies are STI and the use of modern contraceptives [22, 39, 47, 48]. Due to the financial issue and the long distance the suburban residents should travel to visit doctors' offices, and private laboratories, it is essential to equip health centers to meet women’ health needs.

### Strengths and limitations

As the present participants were from suburban areas, one limitation of the present study is the low generalizability of findings to other areas. Also, the COVID-19 pandemic limited women’s visits to the health centers. Thus, and the researchers had to visit the participants’ home two times. The recall bias is considered another limitation of the present research. In addition, the data were completed as self-reports thus women could have provided socially desirable answers. However, the researcher tried to reduce the effects of this bias with an emphasis on the confidentiality of information. Besides the above-mentioned limitations, the present research had a number of strengths. This study was conducted for the first time in the suburban areas of Bandar Abbas. The access variable was addressed separately, which, to the researcher's knowledge, had received less attention in previous studies. The results of this study can help develop educational interventions, with focusing on effective variables compare future related studies.

## Conclusion

To increase utilization of CCS, there is a need to increase women’s access to screening facilities. This should be the first priority of health policy makers. This should be followed by an increase in suburban women’s knowledge of the need to get screened. The present study showed that certain groups of suburban women need more attention or intervention, for example, those living in low SES, the widowed and divorced, and those who smoke. Most importantly, there is a need to make effective and systematic policies and guidelines for CCS among women at risk. Screening and communication approaches should be purposeful and appropriate to the needs of different demographic groups. The present study can help policymakers develop effective strategies for CCS in suburban areas. It is suggested to carry out qualitative studies on the exact barriers to screening in suburban women so that an accurate recognition of barriers can facilitate the development of systematic interventions. It is also suggested to conduct studies on the factors affecting women in high-risk groups, such as sex workers and women who consume tobacco, so that the comparative results of these studies can help identify the key factors involved in the acceptance of CCS in certain groups of society.

## Data Availability

The datasets used and/or analysed during the current study are available from the corresponding author on reasonable request.

## Abbreviations

CCS: Cervical cancer screening
CC: Cervical cancer
