# Correction: Utilization of cervical cancer screening services and its associated factors in Iran: a case–control study

**DOI:** 10.1186/s13027-023-00506-x

**Published:** 2023-04-25

**Authors:** Sara Dadipoor, Azin Alavi, Zainab Kader, Hadi Eshaghi Sani Kakhaki, Shokrollah Mohseni, Nahid Shahabi

**Affiliations:** 1 Mother and Child Welfare Research Center, Hormozgan University of Medical Sciences, Bandar Abbas, Iran; 2 Social Determinants in Health Promotion Research Center, Hormozgan Health Institute, Hormozgan University of Medical Sciences, Bandar Abbas, Iran; 3 The Centre for Interdisciplinary Studies of Children, Families and Society, Faculty of Community and Health Sciences, University of the Western Cape, Bellville, South Africa

**Correction to**: *Infectious Agents and Cancer (2023) 18:17.*

10.1186/s13027-023-00496-w.

Following publication of the original article [1], the authors identified an error in the author name of the third author.

The incorrect author name is: Zeinab Kader.

The correct author name is: Zainab Kader.

The author group has been updated above and the original article [1] has been corrected.
